# Sustainable Management of Voluntary Culling Risk in Primiparous Zaraibi Goats in Egypt: Roles of Season and Reproductive and Milk Production-Related Traits

**DOI:** 10.3390/ani11082342

**Published:** 2021-08-09

**Authors:** Ali Ali El-Raghi, Mahmoud A. E. Hassan, Ibrahim T. El-Ratel, Nesrein M. Hashem, Sameh A. Abdelnour

**Affiliations:** 1 Department of Animal Production, Faculty of Agriculture, Damietta University, Damietta 34517, Egypt; ali21384@yahoo.com; 2 Animal Production Research Institute (APRI), Agriculture Research Center, Ministry of Agriculture, Dokki, Giza 12619, Egypt; M.hassan55213@gmail.com; 3 Department of Poultry Production, Faculty of Agriculture, Damietta University, Damietta 34517, Egypt; ibrahim.talat81@yahoo.com; 4 Department of Animal and Fish Production, Faculty of Agriculture (El-Shatby), Alexandria University, Alexandria 21545, Egypt; 5 Animal Production Department, Faculty of Agriculture, Zagazig University, Zagazig 44511, Egypt

**Keywords:** culling risk, Zaraibi goats, reproductive traits, milk traits, sustainable farm management

## Abstract

**Simple Summary:**

Sustainable production of livestock farms mainly depends on efficient management and precise decisions. One of the most important managemental decisions that play a great role in the continuation of production and profitability is culling. Excluding non-efficient animals from the production cycle is a very critical decision as a wrong and aggressive culling decision may cause a certain loss in farm profitability by increasing costs of replacement. Identifying factors that affect culling risk can aid breeders in creating suitable interventions to maintain productive animals. In this study, logistic regression analysis was used to study the pattern of culling risk factors in primiparous Nubian goats. The effect of ten independent factors including season, reproductive traits, and milk production traits on the percentage of voluntary culling risk was studied. The presented outcomes clearly indicated that birth weight, total litter size at birth, litter size at weaning, number of kids dead, total milk yield, average daily milk yield, lactation period, and age at first kidding had significant effects on culling risk, while both the birth season of the dam and kidding season did not exhibit significant effects on culling risk. In practice, monitoring of traits that indicate milk imbalance and older age at first kidding could promote identifying goats at high risk of being culled.

**Abstract:**

The purpose of the current study is to reconnoiter the relationships between season (birth season, BS, and kidding season, KS), reproductive traits (birth weight, BW; total litter size at birth, TLS; litter size at weaning, LSW; the number of kids dead, NKD, and; age at first kidding, AFK) and milk production (total milk yield, TMY; average daily milk yield, DMY, and; lactation period, LP), with voluntary culling risk in primiparous Zaraibi goats. Records of 637 primiparous does were collected during the period 2008–2014 from a herd of Zaraibi goats raised at the El-Serw Experimental Station, which belongs to the Animal Production Research Institute. Our data revealed that the voluntary and involuntary culling was 89.12% and 10.88%, respectively. Moreover, the BW, TLS, LSW, NKD, TMY, DMY, LP, AFK had significant effects on culling risk (*p* < 0.05), while both the season of birth and kidding did not exhibit significant effects on culling risk. The thinnest goats at birth were more likely to be culled compared to those with body energy reserves. Does with weaned twins and triplets kids were 9.5% (OR = 0.905) and 43% (OR = 0.570), respectively less likely to be culled compared to those with singles. Likewise, does with dead twins and triplets kids had 2.566 and 2.138 times, respectively higher odds of culling compared to those with singles. Interestingly, culling risk decreased with 74.6% (OR = 0.254), 79.8% (OR = 0.202), and 75.6% (OR = 0.244) in does with TMY, LP, and DMY more than 230 kg, 260 days, and 0.800 kg, respectively compared to their counterparts (less than 185 kg, 240 day, and 0.500 kg, respectively). Moreover, culling risk increased steadily along with increasing AFK; the animals with an AFK of more than 24 months had 2.974 times higher odds of culling compared to those with an AFK that varied between 22 and 24 months. It could be concluded that the most critical issues for higher culling probability in dairy goats were attributed to the lower TMY (<185 kg) and DMY (<0.5 kg), and shorter LP (<245 days), as well as older age at first kidding (>2 years). This putative information could be used as indicators to enhance the management and genetic approaches in dairy goats and thus sustain productivity with low cost.

## 1. Introduction

Zaraibi goats (Egyptian Nubian) are one of the utmost efficient and famous local breeds in Egypt and many other countries all over the world [1]. This breed is a dual-purpose breed that is efficient to produce good quality meat and as well as to produce milk in a long lactation season [2]. This breed is broadly dispersed in the Northern Delta, Egypt. Recently, Egyptian animal breeders are attentive to goat breeding for milk production due to the excessive increase in demand for dairy products [3]. The profitability and economic efficiency of goat farms could be optimized by increasing the productive life of each animal. The average age at first kidding (AFK), total liter size (TLS), litter size at weaning (LSW), and total milk yield (TMY) in Zaraibi goats is 691 days, 2.9 kids, 2.5 kids, and 229.02 kg/season respectively, as reported previously [3,4,5]. Currently, the most common critical point to reduce rearing costs is optimizing the culling rate in dairy goat farms. In intensive herds, elevated rates of culling are a burden in various modern farms of dairy goats, particularly those that wish to enlarge their dairy goat herds but face a scarcity of replacement animals. Culling is well-defined as the departure of animals from the herd because of reproductive problems, low milk production, or infection diseases [6,7]. Several dairy goats are culled due to reasons related to poor welfare provoked by infectious disease, udder or teats problems, infertility, feet and legs (lameness), mastitis, and non-conforming to specifications [8]. Commonly, there are two types of culling; (1) voluntary culling happens when the farmer selects to eradicate a healthy-fertile female due to low productivity, while (2) involuntary culling happens when the farmer is enforced to detach productive, profitable, females because of death, injury, infectious disease, infertility, or illness [7,8,9]. Over the last decades, culling reasons have altered. The percentage of culling owing to voluntary causes (e.g., delayed age at first kidding and low milk yield), has decreased and changed into more disease-related causes [8,10]. Involuntary culling is needed to decrease further suffering in individual animals, but a superior percentage of involuntary culling in the herd shows poor animal welfare and ineffective utilization of animal resources, which decreases sustainable dairy production [10,11]. 

Enhancement of culling verdict sustenance tools will support to augment the economically best productive lifespan for specific animals. Furthermore, the extension of a productive lifetime would reduce replacement costs [11]. It is well known that an increment in the longevity of dairy goats is a favorable strategy because it means that the cost of breeding replacements is amortized over a longer period of income production [8,12]. 

Didarkhah et al. [13] reported that the involuntary culling frequency in dairy goats was 12% of the total population. The preceding analysis did not determine the voluntary rate and additional reasons for culling risks in dairy goats. While Malher et al. [14] reported that the voluntary culling rate in dairy goats due to low productivity was 36.6% on average. In another study on dairy goats, Gautam et al. [15] noticed that the daily risk of goats culled that produced 80 kg milk at the first lactation was 0.84 (95%, CI 0.58–1.23) times compared to the daily risk of goats culled that produced 30 kg milk at the first lactation. Generally, the unsuitable body condition score and lowering of individual milk yield breeding values were critical features for high culling rates in dairy goats [11]. The distribution of culled dairy goats in accordance with milk yield, body condition score, age of animals, the season of birth, and parities could help the breeders to reduce the culling associated risk via a more effective, timely decision [16]. Prior scientific records described that high and consistent milk yield could substantially reduce culling rates in dairy herds [17]. 

A good understanding of the main risk factors for culling can be utilized to improve longevity in various dairy herds. Considering this information, it is plausible to explore features that can serve as early indicators of culling in the dairy herds system. Moreover, depending upon how strong the impact of a specific risk issue on elimination is, the right culling decision for an animal from a flock might increase the farm profitability or at least incur minimal loss [8,15]. Since culling is the main issue affecting the sustainability in dairy farms including goat farms, it is of interest to have an understanding of the reasons influencing the culling in Egyptian dairy goats. There is a lack of current investigations in this topic concerning the dairy goat industry, and characterizing the risk reasons for culling in dairy goats is restricted. Despite several studies that identify risk features for culling in dairy cows [6,7,8,10], the published studies in dairy goats are scarce, specifically for Zaraibi goats that are reared under Egyptian management conditions. To address this data gap, this study was devoted to exploring risk factors (season, reproductive traits, and milk production traits) that may affect voluntary culling patterns of dairy goat herds in Egypt. We used an odd ratio (OR) statistical analysis that estimates the strength of the association between two events to clarify the association effects of some risk factors on culling rates. This data will permit managers/breeders of dairy goat herds to pick more concerted tactics for culling.

## 2. Material and Methods

### 2.1. Data Collection

All data used for the current exploration was based on recordings in the Zaraibi herd at the El-Serw Experimental Station, Damietta city, the Northeastern region of the Nile Delta (N0 21, 31°; E0, 45, 31°), Egypt. This farm belongs to the Animal Production Research Institute (APRI), Ministry of Agriculture, Giza, Egypt. Data from a random sample of 637 primiparous Zaraibi does (dairy herd) were collected for this study over seven consecutive years from 1 January 2008 to 31 December 2014. The details of culling causes were obtained from the inventory and death recording registers. Voluntary and involuntary classes of culling reasons were formed by accumulating the parallel culling causes presented in the APRI system. The system used by APRI considers the reasons for voluntary culling based on several issues such as feet, leg, udder or teats problems, low productivity, fertility problems, and non-conforming to specifications, while the reasons for involuntary culling were always infectious diseases. Moreover, the records of studied traits such as birth date, kidding date, birth weight, culling date, kidding season, total litter size at birth, litter size at weaning, number of kids dead, total milk yield, average daily milk yield, and lactation length were also obtained from the APRI recording system. All irregular and incomplete records were excluded from the analysis. 

### 2.2. Animal Management and Feeding

Animals were housed in semi-open pens, and the feed was provided to animals twice daily. The diets were composed of green Egyptian clover (*Trifolium Alexandrinum*) during the winter and spring seasons and clover hay and/or crop stubbles or rice straw during autumn and summer seasons, plus concentrate mixture when available. Diets covered the nutritional requirements for animals according to the National Research Council [18]. Drinking water was available just as mineral blocks. Mating started for the newly presented does around 15 months of age, with an average body weight of 30 kg. Two weeks before the beginning of the mating season, a supplementary concentrate was offered at a rate of about 0.25 kg/doe/day. The supplementary feed was given also during the last 2–4 weeks of pregnancy and over the first week of lactation if it was available. Zaraibi does were randomly divided into mating groups of 25–30 does per fertile buck. To avoid inbreeding, mating bucks were replaced after two mating seasons with others within the flock. Mating was conducted for half of the herd in the autumn (to have kids in the spring), and for the other half in the spring (to have kids in the autumn) [3]. After parturition, kids were ear-tagged and kept with their dams until weaning at 3 months of age. During the suckling period, milk production was induced with an oxytocin injection and stimulated with hand milking as described by Abdel-Gawad and Desoky [19]. 

### 2.3. Statistical Analysis

Potential risk factors (BS, KS, BW, KY, TLS, NKD, LSW, TMY, DMY, LP, and AFK) were included as independent variables in the model. The odds ratio was used as an approximate measure of relative risk. For each culling event, a multivariate logistic regression model was run using PROC logistic [20] in order to examine the relationships with potential risk factors. The model was:

*Log* (*Π*CUL/(1 − *Π*CUL)) = *β*0 + *β*1BS + *β*2 KS + *β*3BW + *β*4 KY + *β*5TLS + *β*6NKD + *β*7LSW + *β*8TMY + *β*9DMY + *β*10LP + *β*11AFK]

where *Π*CUL is the probability of culling, BS is the birth season, KS is the kidding season, BW is the birth weight, KY is the kidding year, TLS is the total litter size, NKD is the number of kids dead, LSW is the litter size weaned, TMY is the total milk yield, DMY is the daily milk yield, LP is the lactation period, and AFK is the age at first kidding. The classes of each studied variable introduced in the statistical model are shown in Table 1. The Pearson correlation coefficient between the potential risk factors was determined through PROC logistic according to the same program mentioned before. A Shapiro-Wilk test was conducted in order to check for normality as described by Razali and Wah [21]. Culling rates were calculated as the number of goats for which culling took place divided by the total number of the studied goats. Survival rates were calculated as the number of non-culled goats divided by the total number of the studied goats. Statistical significance was set at the 0.05 level.

## 3. Results

### 3.1. Descriptive Statistics

As shown in Table 1, the major descriptive statistics for the traits assessed (BS, KS, BW, TLS, LSW, NKD, TMY, LP, DMY, and AFK) with potential effects on culling rate (CR%) in the Egyptian Zaraibi goat population were considered. The mean values of TLS, LSW, and NKD in primiparous dairy Zaraibi goats were 3.157, 1.769, and 1.388, respectively. The averages of BW, TMY, LP, and DMY were 1.706 kg, 204.701 kg, 243.895 days, and 0.840 kg, respectively.

### 3.2. Culling and Survival Rates after First Kidding

The overall culling rate of primiparous goats in the present flock was 36.05% (239/663) (Table 1). The various reasons for voluntary and involuntary culling in primiparous goats during the study period (2008–2014) are shown in Table 2. It was that the proportion of voluntary culling by selling was 8.19 times higher than involuntary culling due to mortality. 

**Figure 1:**
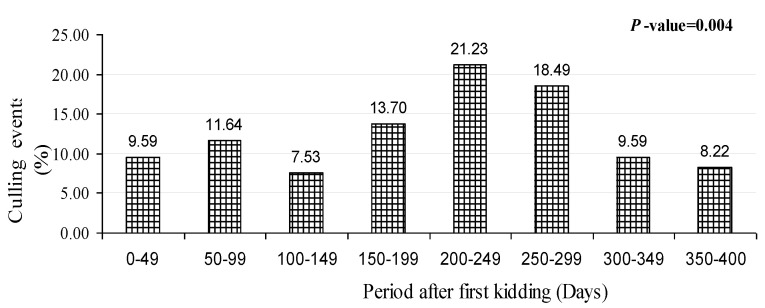
Distribution of culling events in primiparous goats.

Significant differences were shown for the distribution of culling events in primiparous goats (*p* = 0.004) over the different age categories after first kidding (Figure 1). The higher culling rate was detected at 200–249 days (21.23%) after first kidding followed by 250–299 days (18.49%). Our data indicated that the lowest culling events were observed in age categories 100–149 days (7.53%) and 350–400 days (8.22%).

### 3.3. Potential Risk Factors (Season, Birth Weight, Litter Size, Milk Traits, and Age at First Kidding)

**Figure 2:**
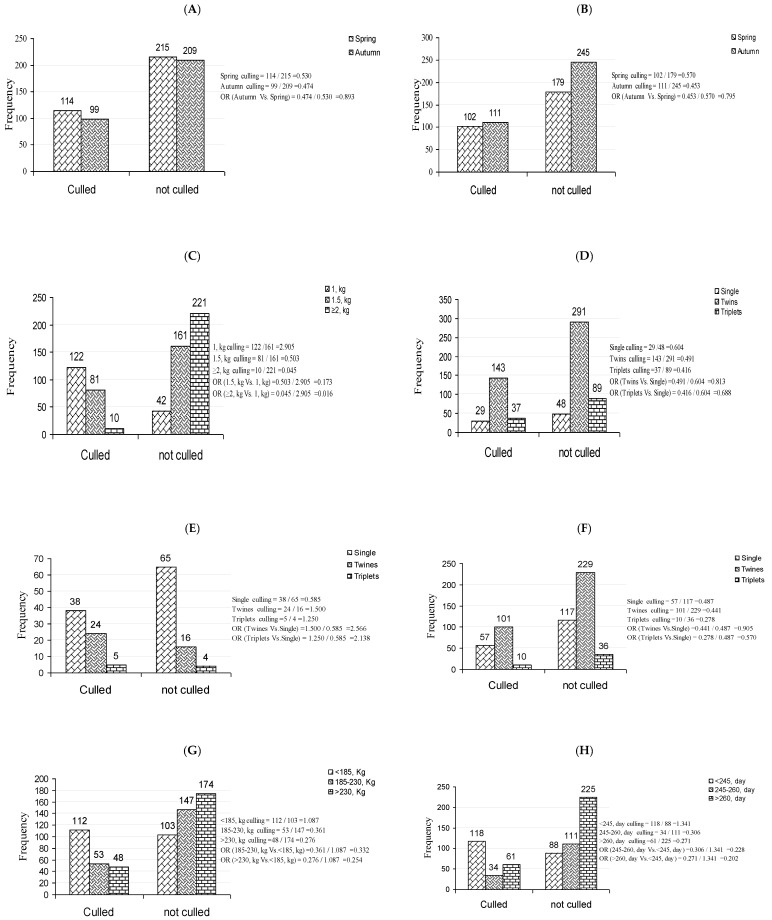

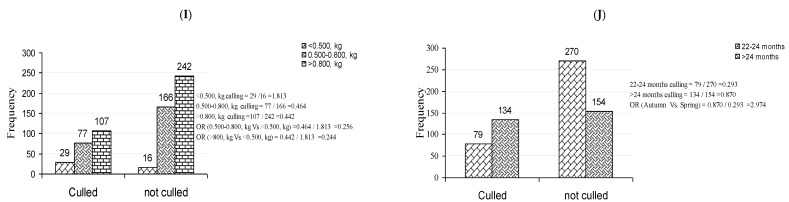
Relationships between culling risk in primiparous goats and birth season (**A**), kidding season (**B**), birth weight (**C**), total litter size (**D**), numbers of kids dead (**E**), numbers of kids weaned (**F**), total milk yield (**G**), length of lactation (**H**), average daily milk yield (**I**), and age at first kidding (**J**).

Table 3 summarizes the logistic regression analysis results of risk factors associated with culling rates in primiparous dairy Zaraibi goats. Numbers of culled and not culled animals are presented in Figure 2A–J. There were significant effects (*p* < 0.05) of body weight at birth, litter size at kidding and weaning, number of kids dead, total milk yield, lactation period, average daily milk yield, and age at first kidding on culling risk, while the seasonality (birth and kidding seasons) did not have significant effects (*p* > 0.05). Primiparous does with body weights at the birth of 1.5 kg and ≥2 kg were 82.7% (OR = 0.173) and 98.4% (OR = 0.016) less likely to be culled compared to those that weighed only 1 kg. The does with weaned twins and triplets kids were 9.5% (OR = 0.905) and 43% (OR = 0.570), respectively less likely to be culled compared to those with singles. Likewise, the animals with dead twins and triplets kids had 2.566 and 2.138 times, respectively higher odds of culling compared to those with singles. Considering milk production, there were significant decreases in culling risk (66.8%; OR = 0.332 and 74.6%; OR = 0.254) for goats with a total milk yield varying between 185 and 230 kg and those producing more than 230 kg, respectively compared to individuals with a total milk yield on average lower than 185 kg. Furthermore, goats with average daily milk yield from 0.500 to 0.800 kg and those with more than 0.800 kg were 74.4% (OR = 0.256) and 75.6% (OR = 0.244), respectively less likely to be culled compared to those with an average daily yield lower than 0.500 kg. Culling risk decreased by 77.2% (OR = 0.228) and 79.8% (OR = 0.202) for goats with a lactation length varying between 245 and 260 days and of more than 260 days, respectively compared to those with a lactation length below 245 days. The younger a goat at first kidding, the lower the probability of culling; in comparison to the animals with a first kidding age varying between 22 and 24 months, the animals with a first kidding age of more than 24 months had 2.974 times higher odds of culling.

### 3.4. Correlation Coefficient between Different Factors Involved in Culling Risk

In the present study, Spearman’s rank correlation coefficient was used to determine the association between the different factors involved in culling risk in dairy goats (Table 4). Interestingly, there were significant and positive correlation coefficients between all potential risk factors except for the associations between TMY—NKD, LSW—NKD, NKD—AFK, NKD—BW, and AFK–BW that were negative.

## 4. Discussion

Culling is a major cost for goat farms but also an indispensable part of managing herd productivity. This study aimed to identify the culling rates of Egyptian dairy goats and to classify the reasons and risks for culling. The overall culling rate of dairy goats was 89.12% for voluntary culling and 10.88%, for involuntary culling. The investigated traits; BW, TLS, LSW, NKD, TMY, DMY, LP, and AFK had significant effects on culling risk (CR; *p* < 0.05), while both birth and kidding seasons did not exhibit any significant effects (*p* > 0.05) on CR. Dairy goats with weaned twin and triplet kids had lower CR (9.5% and 53%, respectively) than those with singles; we conclude that the most critical issues for higher culling probability in dairy goats were lower TMY (<185 kg), DMY (<0.5 kg), and shorter LP (<245 days), as well as older age at first kidding (>2 years). This putative information could enhance the management approaches in dairy goats and increase productivity with lower costs. This is the first study that tried to identify the potential risks of culling in dairy goats under the Egyptian management system. 

Under normal circumstances, replacement rates are decreased, and higher profitability of goats is gained by increasing longevity of female goats which would lead to higher profitability in the dairy goat industry [15]. Didarkhah et al. [13] clarified that the involuntary culling rate in dairy goats was 12%. The previous study did not demonstrate the voluntary rate and further reasons for culling risks. Ghaderi-Zefrehei et al. [22] reported that 27.11% of Holstein dairy cows were culled voluntarily. Our data reported that the most significant motive for voluntary culling was body condition score followed by lower milk production. Moreover, Chiumia et al. [17] found that the herd culling rate was 33.7% and this result is consistent with the findings of Rilanto et al. [8], who demonstrated that the overall culling rate of Estonian dairy cows was 26.24%. 

Rilanto et al. [8] reported that the most common reasons farmers stated for the culling of dairy cows were feet/claw disorders (26.4%), udder disorders (22.6%), metabolic and digestive disorders (18.1%), and fertility problems (12.5%). In the current study, we found that a lower milk production and body condition score were the most common causes for culling in the dairy goat industry. In line with our results, the study of [8] demonstrated that lower milk yield was associated with greater culling hazards during lactation. Moreover, dairy cows from larger herds and/or herds with decreasing size and higher milk yields had a higher culling probability [8]. It was reported that the main motives for voluntary culling were udder complications (26.9%) and reproductive problems (27.4%) in dairy cows [17]. In line with our results, Malher et al. [14] reported that voluntary culling in dairy goats due to low productivity was 36.6%. According to animal age categories, the most common time for culling was detected at earlier ages (1–2 years; 23.8%), and at this age category, the culling rate due to infertility disorders was 17.1 %. Furthermore, infertility problems were reflected as the only reason or as the second reason associated with low production or other voluntary culling reasons [14].

In dairy goats, an unsuitable body condition score and lowering of individual milk yield breeding values were critical features for high culling rates. Distribution of culled dairy goats according to stages of animal ages, body condition score, milk yield, the season of birth, and parities could help the breeders to reduce the culling associated risks by making more effective and timely decisions [16]. 

Low milk production and older age at first kidding were the most frequently described codes for dairy goats being culled. We observed that goats that had lower milk production (<185), shorter LP (<245 days) and average daily milk (<0.5 kg), and older age at first kidding (>2 years) were more frequently culled from the herd. Similar to our findings, [11] reported that cows that became pregnant later in lactation had an augmented risk of culling in the following lactation. Low milk yield is a noticeable jeopardy issue for culling. To keep or enhance herd productivity, dairy farmers will make an economic decision to cull cows producing below a certain threshold and replace them with higher producing cows. Previous works reported that a higher and consistent milk yield could substantially reduce culling rates in dairy herds [11,23]. The rate of culling tended to decrease with an increasing number of TLS, as twinning is considered one of the advantages of breeding goats specifically under intensive farming systems. Contradictory results were reported by both Bicalho et al. [24] and Probo et al. [25] in dairy herds, they reported that cows with twins had higher culling rates compared to cows with single calves as twinning is considered an undesirable trait in large animals.

Likewise, the AFK was another significant factor affecting voluntary culling risk. Goats with longer AFK (>2 years) had 2.974 times higher odds of culling compared to those with kidding ages in the optimum period (between 22 and 24 months). These findings are in line with those observed for heifers. Heifers that calved after 30 months of age were 5.52 times more likely to be culled within 50 days after first calving compared to heifers that calved before 22 months of age (*p* < 0.001) [26].

These differences, regardless of the animals’, species, could be attributed to poor management, reproductive disorders, and effects of diseases on the body conformation during the period of growth and puberty. Usually, culled cows had a greater number of services per conception during life, a greater interval to the first service, and increased days open [27]. Bach [28] showed that the heifers that needed two inseminations to conceive were 26% less likely to complete their first lactation compared to the heifers that needed only one insemination, and the probability of survival gradually decreased as the number of services increased. Moreover, Fodor et al. [26] indicated that culling risk increased steadily along with increasing AFK. However, very few studies have characterized the risk and reasons for culling in the dairy goat industry. Thus, understanding the risk factors for the culling of dairy goats such as reproductive attributes, diseases, lower TMY or AFK in dairy goats is essential for enhancing the sustainability of production for this breed. 

In many dairy herds, the main reasons for culling are low milk production, mastitis, and infertility [29]. In high-producing animals, Vergara et al. [30] showed that 16–32% of the culling rates could be attributed to reproductive complications, which lead to excessive herd replacement costs. Moreover, the study carried out by Gautam et al. [15] on dairy goats revealed that the involuntary losses may be reduced if an elevated milk solids yield in the first lactation of does is favorably achieved from 2 years. 

Evidence suggests that the higher culling risk in does having two or three dead kids might be ascribed to the reduction of milk production or the milk is not covering the requirements of kids during the suckling period. Despite the development of a selective policy for newborn management, more study is desirable to detect more precise predictors of deficiency and insufficient milk production for newborn kids over the suckling period. So, increasing the productive lifespan for goats will at some point decline the average age at death per born kid. 

Collectively, the results of the present study suggest that the regular monitoring of daily milk yield, body weight at birth, and age at first kidding could support the detection of goats at high risk of being culled. Age of puberty and sexual maturation in goats could affect the age at first mating and thus affect the age at first kidding [31]. Besides, kidding for the first time at an older age was related to an increase in the voluntary culling rate in goats. In line with other amendments, it is important to pay attention to disposing situations that might be associated with the incidence of stillbirth or death during the weaning period, as these are important risk factors for culling and longevity of dairy goats [32,33]. Additionally, higher culling risk due to AFK (>2 years) indicates that the reproductive traits of a goat might be an important risk factor for culling due to the increased probability of a reproductive disorder. This is identified to be accompanied by an increased mortality hazard in goats as well as an increased risk of post-partum diseases eventually leading to culling [15]. Good feeding practices would enhance body condition score and energy balance during the lactation period [34] and thus improve the productive and reproductive aspects along with the life span as well as reducing the culling risk in the goat industry [12,17,35,36]. Therefore, through consistent monitoring of the traits that properly indicate feeding and lactating dairy goats, the risk of voluntary culling can be minimized by preventing lowered milk yield, delayed age at first kidding, and decreasing the mortality of kids during the weaning period. 

## 5. Conclusions

This study identified for the first time the predisposing aspects for increased risk of voluntary and involuntary culling in Primiparous Zaraibi goats in Egypt. The overall culling rate of dairy goats was 36.05%. Furthermore, this present analysis concluded that the most critical issues for higher culling probability in dairy goats were attributed to the low TMY (<185 kg) and DMY (<0.5 kg), and short LP (<245 days), as well as an older age at first kidding (>2 years). This putative information could enhance the management approaches in dairy goats and increase productivity with lower costs. Assuming successful farm practices to improve TMY is required to decrease the mortality in suckling kids and reduce the economic losses due to a higher voluntary culling rate. Moreover, improvements in body conformation, nutritional, and fertility aspects will decrease involuntary culling and permit for more culling decision-making on functionally sound animals. Decision-making concerning the productive life of dairy goats is currently more art than science and may help optimize the productive life of dairy goats. In practice, monitoring of traits that indicate milk imbalance and older age at first kidding could promote identifying goats at high risk of being culled. In addition, this scheme may contribute to decreasing the associated risk by making more effective and timely decisions in the dairy goat industry.

## Figures and Tables

**Table 1:** Description of variables used to study the risk factors for culling rate in primiparous dairy Zaraibi goats.

Variable	n	Mean	S.D.	Classes for Statistical Analyses
Birth season (spring or autumn)	637	-	-	1, 2
Kidding season (spring or autumn)	637	-	-	1, 2
Birth weight	637	1.706	0.300	1, 1.5, ≥2
Total litter size	637	3.157	0.558	1, 2, 3
Number of kids weaned	637	1.769	0.593	1, 2, 3
Number of kids dead	637	1.388	0.620	1, 2, 3
Total milk yield (kg)	637	204.701	63.135	<185, 185–230, >230
Lactation period (days)	637	243.895	48.292	<245, 245–260, >260
Daily milk yield (kg)	637	0.840	0.218	<0.500, 0.500–0.800, >0.800
Overall culling rate (%)	239	36.05	-	0, 1
Survival rate (%)	424	63.95	-	0, 1

**Table 2:** Distributions of primiparous goats according to the different culling reasons.

Type of Culling	Causes	*n*	(%)
Voluntary	Feet, leg, udder or teats problems, low productivity, fertility problems, non-conforming to specifications	213	89.12
Involuntary	Infectious diseases	26	10.88
Total	-	239	100

**Table 3:** Logistic regression analysis of different factors associated with the probability of being culled after first parity in primiparous dairy Zaraibi goats.

Items	n	β	S.E.(β)	OR	95% CI	*p*-Value
*Birth season*						
Spring	329			*Ref.*		
Autumn	308	−0.120	0.172	0.893	0.632–1.243	0.304
*Kidding season*						
Spring	281			*Ref.*		
Autumn	356	−0.233	0.172	0.795	0.564–1.111	0.537
*Birth weight (BW, kg)*						
1	164			*Ref.*		
1.5	242	−0.771	0.527	0.173	0.065–0.299	0.001
≥2	231	−1.292	0.533	0.016	0.006–0.119	
*Total litter size (TLS)*						
Single	77			*Ref.*		
Twins	434	−0.129	0.232	0.813 *	0.722–1.794	0.001
Triplets	126	−0.314	0.319	0.688	0.732–2.562	
*Number of kids dead*						
One	103			*Ref.*		
Two	40	0.942	0.381	2.566 **	1.214–4.424	0.001
Three	9	0.759	0.701	2.138	1.541–3.451	
*Litter size at weaning (LSW, kid)*						
Single	174			*Ref.*		
Twins	330	−0.099	0.200	0.905	0.611–1.342	0.001
Triplets	46	−0.561	0.392	0.570	0.264–1.230	
*Total milk yield (TMY, Kg)*						
<185	215			*Ref.*		
185–230	200	−1.312	0.220	0.332	0.175–0.415	0.001
>230	222	−1.745	0.240	0.254	0.109–0.280	
*Lactation period (LP, days)*						
<245	206			*Ref.*		
245–260	145	−1.514	0.253	0.228	0.122–0.362	0.001
>260	286	−1.611	0.204	0.202	0.134–0.298	
*Daily milk yield (DMY, Kg)*						
<0.500	45			*Ref.*		
0.500–0.800	243	−1.570	0.380	0.256	0.099–0.438	0.001
>0.800	349	−1.544	0.369	0.244	0.103–0.440	
*Age at first kidding (months)*						
22–24	349			*Ref.*		
>24	288	1.443	0.637	2.974	1.821–3.336	0.001
*Kidding year (linear trend)*	637	−1.318	0.336	0.604 ***	0.411–0.983	0.047

β, regression coefficient; S.E.(β)., standard error; OR, odds ratio; CI, confidence interval; Ref., reference. * E.g. goats with a twins litter size were 18.7% (OR = 0.813; CL = 0.722–1.794) less likely to be culled compared to those with singles. ** E.g. goats with dead twins kids had 2.566 times (OR = 2.566; CL = 1.214–4.424) higher odds of culling compared to those with singles. *** The probability of voluntary culling decreased 39.6% per year (OR = 0.604; CL = 0.411–0.983).

**Table 4:** Correlation coefficients between different factors related to culling risk in primiparous dairy Zaraibi goats.

	TLS	LSW	NKD	AFK	BW
TMY	0.836 ***	0.788 ***	−0.517 **	0.635 **	0.752 ***
TLS	1	0.706 ***	0.517 **	0.569 **	0.863 ***
LSW		1	−0.093 ^NS^	0.664 **	0.633 **
NKD			1	−0.332 ^NS^	−0.439 ^NS^
AFK				1	−0.587 *
BW					1

TMY, total milk yield; TLS, total litter size; LSW, litter size at weaning; NKD, number of kids dead; AFK, age at first kidding; BW, birth weight; * *p* < 0.05; ** *p* < 0.01; *** *p* < 0.001; ^NS^ non-significant.

## Data Availability

Data are confidential and its availability returns to the authors permission.

## References

[1] Capote J., Kukovics S. (2016). Environments and goats around the world: Importance of genetic and management factors. Sustainable Goat Breeding and Goat Farming in Central and Eastern European Countries.

[2] Kholif A.E., Gouda G.A., Hamdon H.A. (2020). Performance and Milk Composition of Nubian Goats as Affected by Increasing Level of Nannochloropsis oculata Microalgae. Animals.

[3] Moawed S.A., Shalaby N.A. (2018). Statistical models for genetic evaluation of some first kidding and lifetime traits of the Egyptian Zaraibi goats. Small Rumin. Res..

[4] Marai I.F.M., Abou-Fandoud E.I., Daader A.H., Abu-Ella A.A. (2002). Reproductive doe traits of the Nubian (Zaraibi) goats in Egypt. Small Rumin. Res..

[5] Soltan Y., Morsy A., Hashem N., Sallam S. (2021). Boswellia sacra resin as a phytogenic feed supplement to enhance ruminal fermentation, milk yield, and metabolic energy status of early lactating goats. Anim. Feed Sci. Technol..

[6] Hadley G.L., Wolf C.A., Harsh S.B. (2006). Dairy Cattle Culling Patterns, Explanations, and Implications. J. Dairy Sci..

[7] Weigel K.A., Palmer R.W., Caraviello D.Z. (2003). Investigation of Factors Affecting Voluntary and Involuntary Culling in Expanding Dairy Herds in Wisconsin using Survival Analysis. J. Dairy Sci..

[8] Rilanto T., Reimus K., Orro T., Emanuelson U., Viltrop A., Mõtus K. (2020). Culling reasons and risk factors in Estonian dairy cows. BMC Vet. Res..

[9] Bell M.J., Wall E., Russell G., Roberts D.J., Simm G. (2010). Risk factors for culling in Holstein-Friesian dairy cows. Vet. Rec..

[10] Ahlman T., Berglund B., Rydhmer L., Strandberg E. (2011). Culling reasons in organic and conventional dairy herds and genotype by environment interaction for longevity. J. Dairy Sci..

[11] Pinedo P., De Vries A., Webb D. (2010). Dynamics of culling risk with disposal codes reported by Dairy Herd Improvement dairy herds. J. Dairy Sci..

[12] Oishi K., Kahi A.K., Nagura Y., Fujita M., Hirooka H. (2008). Effect of culling age of does on milk and meat production in Japanese-Saanen goats. Livest. Sci..

[13] Didarkhah M., Vatandoost M., Dirandeh E., Dadashpour Davachi N. (2019). Characterization and Pattern of Culling in Goats. Arch. Razi Inst..

[14] Malher X., Seegers H., Beaudeau F. (2001). Culling and mortality in large dairy goat herds managed under intensive conditions in western France. Livest. Prod. Sci..

[15] Gautam M., Stevenson M.A., Lopez-Villalobos N., McLean V. (2017). Risk Factors for Culling, Sales and Deaths in New Zealand Dairy Goat Herds, 2000–2009. Front. Vet. Sci..

[16] De Vries A., Marcondes M.I. (2020). Review: Overview of factors affecting productive lifespan of dairy cows. Animal.

[17] Chiumia D., Chagunda M.G.G., Macrae A.I., Roberts D.J. (2012). Predisposing factors for involuntary culling in Holstein–Friesian dairy cows. J. Dairy Res..

[18] National Research Council (U.S.), Committee on Nutrient Requirements of Small Ruminants, National Research Council, Committee on the Nutrient Requirements of Small Ruminants, Board on Agriculture and Natural Resources, Division on Earth and Life Studies (2007). Nutrient Requirements of Small Ruminants: Sheep, Goats, Cervids, and New World Camelids.

[19] Abdel-Gawad A., Desoky A. (2018). Suckling milk yield of Zaraibi goats as affected by measuring methods. J. Anim. Poult. Prod..

[20] Stokes M.E., Davis C.S., Koch G.G. (2012). Categorical Data Analysis Using SAS.

[21] Razali N.M., Wah Y.B. (2011). Power comparisons of shapiro-wilk, kolmogorov-smirnov, lilliefors and anderson-darling tests. J. Stat. Modeling Anal..

[22] Ghaderi-Zefrehei M., Rabbanikhah E., Baneh H., Peters S.O., Imumorin I.G. (2017). Analysis of culling records and estimation of genetic parameters for longevity and some production traits in Holstein dairy cattle. J. Appl. Anim. Res..

[23] Gröhn Y.T., Eicker S.W., Ducrocq V., Hertl J.A. (1998). Effect of Diseases on the Culling of Holstein Dairy Cows in New York State. J. Dairy Sci..

[24] Bicalho R.C., Vokey F., Erb H.N., Guard C.L. (2007). Visual Locomotion Scoring in the First Seventy Days in Milk: Impact on Pregnancy and Survival. J. Dairy Sci..

[25] Probo M., Pascottini O.B., LeBlanc S., Opsomer G., Hostens M. (2018). Association between metabolic diseases and the culling risk of high-yielding dairy cows in a transition management facility using survival and decision tree analysis. J. Dairy Sci..

[26] Fodor I., Lang Z., Ózsvári L. (2020). Relationship of dairy heifer reproduction with survival to first calving, milk yield and culling risk in the first lactation. Asian-Australas J. Anim. Sci..

[27] Millan-Suazo F., Erb H.N., Smith R.D. (1989). Risk factors for reason-specific culling of dairy cows. Prev. Vet. Med..

[28] Bach A. (2011). Associations between several aspects of heifer development and dairy cow survivability to second lactation. J. Dairy Sci..

[29] Caraviello D., Weigel K., Gianola D. (2004). Prediction of longevity breeding values for US Holstein sires using survival analysis methodology. J. Dairy Sci..

[30] Vergara O., Elzo M., Cerón-Muñoz M. (2009). Genetic parameters and genetic trends for age at first calving and calving interval in an Angus-Blanco Orejinegro-Zebu multibreed cattle population in Colombia. Livest. Sci..

[31] Silpa M., Naicy T., Aravindakshan T., Radhika G., Joan J., Jinty S. (2020). Ovarian expression, polymorphism identification and association of SIRT3 gene with reproduction traits in goats. Anim. Biotechnol..

[32] Castañeda-Bustos V.J., Montaldo H.H., Torres-Hernández G., Pérez-Elizalde S., Valencia-Posadas M., Hernández-Mendo O., Shepard L. (2014). Estimation of genetic parameters for productive life, reproduction, and milk-production traits in US dairy goats. J. Dairy Sci..

[33] Pérez-Razo M., Sánchez F., Torres-Hernández G., Becerril-Pérez C., Gallegos-Sánchez J., González-Cosıío F., Meza-Herrera C. (2004). Risk factors associated with dairy goats stayability. Livest. Prod. Sci..

[34] Hashem N.M., El-Zarkouny S.Z. (2017). Metabolic attributes, milk production and ovarian activity of ewes supplemented with a soluble sugar or a protected-fat as different energy sources during postpartum period. Ann. Anim. Sci..

[35] Guerrero A., Sañudo C., Campo M.M., Olleta J.L., Muela E., Macedo R.M.G., Macedo F.A.F. (2018). Effect of linseed supplementation level and feeding duration on performance, carcass and meat quality of cull ewes. Small Rumin. Res..

[36] Zamuner F., DiGiacomo K., Cameron A., Leury B. (2020). Effects of month of kidding, parity number, and litter size on milk yield of commercial dairy goats in Australia. J. Dairy Sci..

